# Extreme Heat and Pregnancy Outcomes: A Scoping Review of the Epidemiological Evidence

**DOI:** 10.3390/ijerph19042412

**Published:** 2022-02-19

**Authors:** Sarah Syed, Tracey L. O’Sullivan, Karen P. Phillips

**Affiliations:** Interdisciplinary School of Health Sciences, Faculty of Health Sciences, University of Ottawa, Ottawa, ON K1N 6N5, Canada; ssyed020@uottawa.ca (S.S.); tosulliv@uottawa.ca (T.L.O.)

**Keywords:** extreme heat, pregnancy, climate change, environmental exposure, premature birth, pregnancy outcome

## Abstract

Background: Extreme heat caused by climate change is a major public health concern, disproportionately affecting poor and racialized communities. Gestational heat exposure is a well-established teratogen in animal studies, with a growing body of literature suggesting human pregnancies are similarly at risk. Characterization of extreme heat as a pregnancy risk is problematic due to nonstandard definitions of heat waves, and variable study designs. To better focus future research in this area, we conducted a scoping review to assess the effects of extreme heat on pregnancy outcomes. Methods: A scoping review of epidemiological studies investigating gestational heat-exposure and published 2010 and 2020, was conducted with an emphasis on study design, gestational windows of sensitivity, adverse pregnancy outcomes and characterization of environmental temperatures. Results: A sample of 84 studies was identified, predominantly set in high-income countries. Preterm birth, birthweight, congenital anomalies and stillbirth were the most common pregnancy outcome variables. Studies reported race/ethnicity and/or socioeconomic variables, however these were not always emphasized in the analysis. Conclusion: Use of precise temperature data by most studies avoided pitfalls of imprecise, regional definitions of heat waves, however inconsistent study design, and exposure windows are a significant challenge to systematic evaluation of this literature. Despite the high risk of extreme heat events and limited mitigation strategies in the global south, there is a significant gap in the epidemiological literature from these regions. Greater consistency in study design and exposure windows would enhance the rigor of this field.

## 1. Introduction

Climate change increases global temperatures, augments normal seasonal temperatures and increases the frequency and duration of extreme weather events such as heat waves in some regions such as Europe (2003, 2015), Russia (2010), Buenos Aires (2013), North America (2021) [1,2]. There is no globally-accepted definition for heat waves, though generally heat waves are declared when ambient temperature exceeds a threshold above normal high temperatures, sustained for a minimum number of days or weeks [3,4]. Human health effects occur following sustained exposure to temperatures at the extremes (very high or very low) of normal climactic patterns for a particular region. 

Sustained exposure to high ambient temperatures causes both acute respiratory and cardiovascular health issues, which may lead to death [5]. Heat waves experienced by Europeans in 2003 caused more than 70,000 deaths, generally attributed to heat stress/shock, respiratory/cardiovascular shock or exacerbations of underlying pathologies [6]. Extreme heat contributes to agricultural and ecological drought conditions [1], which in turn contributes to food insecurity, compromises water quality and increases insect-borne diseases [2]. Communities in low to middle-income countries may be disproportionately affected by extreme heat due to food supply issues caused by agricultural impacts and also by more directly adverse health effects in the absence of environmental mitigation strategies [2]. 

Sustained exposure to high ambient temperatures causes both acute heat stress in pregnant people, however, increasingly it adversely affects fetal development [7,8]. In humans, a maternal core body temperature of 39 °C is established as a teratogenic threshold; associated with increased developmental risks to the fetus [9]. Poor and racialized communities typically exhibit disparities in maternal and child health [10], further amplified by issues of environmental injustices such as extreme heat [2]. Several systematic reviews have concluded that high ambient temperature is associated with preterm birth, low birthweight and stillbirth [11,12,13]. Confounding the interpretation of this body of literature is varied windows of exposure (e.g., all or part of gestation), inconsistent definitions of excess heat or heat waves, and diverse study designs and populations. 

As climate change-related extreme heat events are likely to continue to increase in frequency and severity [1], with concomitant impacts on local agricultures and ecologies, clarification of the sensitivity to gestational heat exposure is needed. Building on Arksey and O’Malley’s methodological framework [14], a scoping review was conducted to assess the effects of extreme heat on pregnancy outcomes to better focus future research in this field.

## 2. Materials and Methods

### 2.1. Eligibility Criteria 

The main research objective was to identify the major adverse pregnancy outcomes examined in the context of ambient gestational heat exposure, with emphasis on study design, gestational windows of sensitivity, and characterization of environmental temperatures. 

We included empirical, quantitative articles that evaluated the association between ambient gestational heat exposure and pregnancy outcomes. Inclusion criteria consisted of: (i) English-language publications, (ii) publication date between 2010 and 2020; (3) articles reporting live birth (full term or preterm birth), stillbirth, spontaneous abortion/miscarriage, PROM (premature rupture membrane), birthweight, congenital anomalies as variables of interest. Our exclusion criteria included: (i) animal studies; (ii) indoor, workplace or yoga/hot tub evaluated as exposure sources of ambient heat; (iii) outcome variables of interest other than acute pregnancy outcomes (e.g., maternal health outcomes, infant/childhood health). Studies that were not published in English were excluded, as well as articles with restricted access to full text. 

### 2.2. Information Sources and Search Strategy

We conducted a literature review, with the support of a health sciences librarian, using four databases: CINAHL, EMBASE, Medline Ovid, and Scopus. The following is a list of the keywords used in the search: Heat wave; tmax; heat injury; overheat; heat stress; extreme hot weather; reproductive health; maternal health; pregnancy complications; pregnancy outcome; birth weight; stillbirth, pregnancy; fetus; gestational weight gain; miscarriage; preterm birth; congenital disorder; placenta; and maternal heat exposure. Boolean operators were used to group MeSH terms, and keywords expanded to amplify the search. 

### 2.3. Study Selection Process

The search findings and inclusion/exclusion criteria were imported into Covidence^TM^ (Melbourne, Australia) for review. We commenced the study selection process with two reviewers conducting independent reviews of titles/abstracts, followed by full text review [14]. At each screening step, inter-rater discrepancies were resolved by discussion. 

### 2.4. Extraction and Synthesis

Extraction of full text articles was performed in Covidence^TM^ with summary data further extracted to Excel^TM^ software. Briefly, a data extraction template was constructed in Covidence^TM,^ which included title, study setting, design, aim, dates of data collection, participants (number, description), inclusion/exclusion criteria, temperature exposure parameters, pregnancy windows of exposure, pregnancy outcome variables, major results, race/ethnicity and socioeconomic variables collected. We report the minimum incremental temperature measurements obtained from climate monitoring stations/records for each study, noting that many studies aggregated these data for statistical analysis. Exposure to environmental temperature was classified as all or part of gestation, which we defined as: 1st trimester (weeks 1 to 13); 2nd trimester (weeks 14 to 27); or 3rd trimester (weeks 28 to 40+). The synthesis phase of our scoping review consisted of the Covidence^TM^-generated PRISMA flowchart describing the search and screening of articles, followed by quantitative analysis of extracted study characteristics. 

## 3. Results

### 3.1. Literature Search

**Figure 1:**
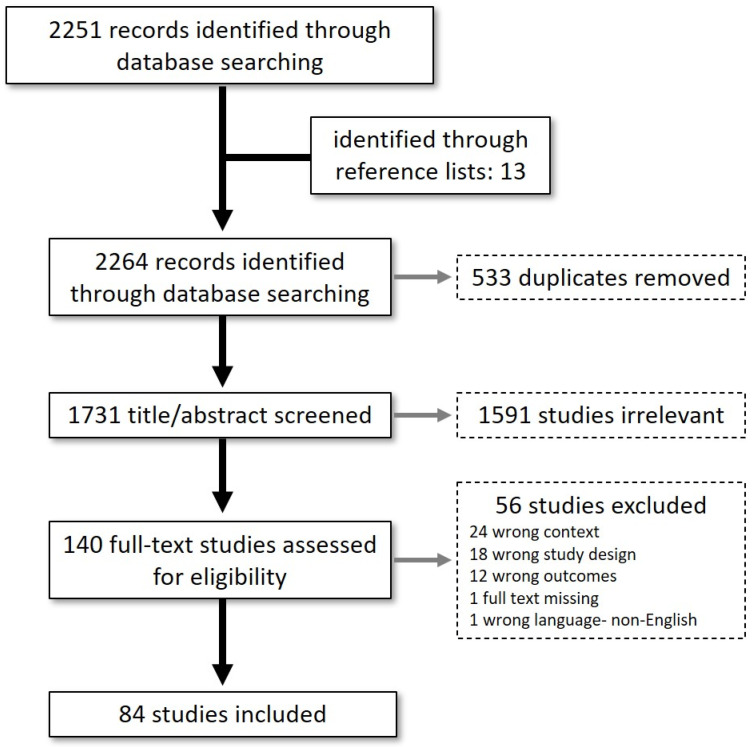
Study Selection Process. Two reviewers performed title/abstract and full-text screening. “Wrong” is used to indicate study characteristics (context, study design, or outcomes), which did not match our inclusion criteria. These unmet criteria were not apparent during abstract screening although were evident upon full text assessment.

The database search yielded in 2251 citations with an additional 13 articles identified manually from published reference lists (Figure 1). Title/abstract screening was performed on 1731 articles after removal of 533 duplicates. A total of 140 full-text articles were assessed for eligibility after the remaining articles were excluded as non-human, non-environmental heat exposures, and non-empirical studies. After further excluding 56 additional studies for failing to meet inclusion criteria (Figure 1), 84 studies [15,16,17,18,19,20,21,22,23,24,25,26,27,28,29,30,31,32,33,34,35,36,37,38,39,40,41,42,43,44,45,46,47,48,49,50,51,52,53,54,55,56,57,58,59,60,61,62,63,64,65,66,67,68,69,70,71,72,73,74,75,76,77,78,79,80,81,82,83,84,85,86,87,88,89,90,91,92,93,94,95,96,97,98] proceeded to the data extraction phase. 

### 3.2. Study Characteristics

The 84 papers were published between 2010 to 2020, as per our inclusion criteria, however the collected/included data in these studies ranged from 1931 to 2020 (Table 1). Most papers analyzed American (29), Chinese (12), Australian (6) or Canadian (5) datasets. The reported study sample sizes ranged from 138 to 56 million, with 40 articles including a dataset of less than 100,000, 26 articles comprised of 100,000 to 1 million datapoints, and 16 studies with a dataset of over one million. Consistent with our inclusion criteria, studies were designed to assess the relationship between gestational exposures to environmental heat and pregnancy outcomes, usually analyzed by regression models or survival analysis/time series (Table 2). Cohort study design was most typical (31), followed by population registry (16), time-series, registry (13) and case-crossover (11). Data relating to race/ethnicity was collected/reported in 35 articles, with socioeconomic variables such as maternal education, housing status and/or income reported for 45 articles. 

Environmental climate parameters were obtained from regional monitoring stations most often in daily (63) or hourly (7) increments. Gestational exposure windows were selected to align with periods of morphogenesis/organogenesis (1st trimester), timing of delivery (3rd trimester or delivery date), or any part of pregnancy to evaluate both acute and chronic temperature stressors. Most articles (61) were designed to evaluate the effects of gestational heat exposure on a single pregnancy outcome, with the remaining studies discussing multiple outcomes. Preterm birth (44), birthweight (21), congenital anomaly (11) and stillbirth (10) were the most common outcome variables, discussed below.

### 3.3. Preterm Birth

Preterm birth was identified as a major outcome variable in 44 articles (Table 3 [17,19,20,24,25,28,31,33,35,37,42,43,44,45,48,52,54,55,57,58,63,64,66,67,71,72,73,76,79,80,81,83,84,85,86,87,88,90,91,92,93,96,97,98]). Most studies implemented a cohort (17), time-series, registry (11) or population registry (10) design, and described positive associations (40) between environmental heat exposures and preterm birth. Although exposure models included many stages of pregnancy, most studies evaluated preterm birth following ambient heat exposure during days/month prior to delivery (2nd–3rd trimester) or the delivery date itself.

### 3.4. Birthweight

Birthweight studies primarily incorporated a cohort design (10) and evaluated heat exposure throughout gestation (Table 4 [16,32,34,39,40,41,47,49,50,54,56,59,61,65,66,68,69,76,91,92,94]). Most studies (14) included both preterm and term births in their analyses. Birthweight was evaluated both as a continuous variable, and as a binary adverse outcome—low birthweight (LBW) < 2500 g. Almost all studies (16) reported that maternal ambient heat exposure reduced birthweight.

### 3.5. Congenital Anomaly

Congenital anomalies included several heterogeneous categories: cardiac defects (5); neural tube defects (2) and orofacial clefts (1); with hypospadias (1) evaluated as a specific outcome variable and two articles examining multiple anomalies (Table 5 [15,22,23,29,53,60,74,75,78,82,95]). Most articles (10) used first trimester maternal heat exposures in their modelling with a case-control/crossover (8) or cohort study design (3). The major category of congenital anomalies—cardiac defects—is heterogeneous, with atrial septal defects, ventricular septal defects and artery transposition as outcome variables. Atrial/ventricular septal defects were positively associated with heat exposure, reported in all five papers examining cardiac congenital anomalies.

### 3.6. Stillbirth

Ten articles included stillbirth as an outcome variable, with most studies (5) using maternal heat exposure during any part of pregnancy for exposure modelling (Table 6 [18,21,30,40,51,57,70,79,87,91]). A range of study designs were employed. All studies reported positive associations between maternal heat exposure and increased risk of stillbirth.

## 4. Discussion

Our scoping review of 84 empirical, peer-reviewed articles, published between 2010 and 2020, yielded a comprehensive evaluation of the effects of gestational ambient heat exposure and pregnancy outcomes. This review emphasized aspects of study design- features, which fundamentally inform the interpretation of the findings and moreover, the comparability of studies for the purposes of meta-analysis or systematic reviews. We did not limit this review to a specific pregnancy outcome, and did not examine the morbidity or mortality of the pregnant people included in these studies. In general, the body of literature reviewed here described findings from birth or hospital registries with substantial sample sizes, using recorded pregnancy outcomes with established definitions [99] or ICD (International Statistical Classification of Diseases and Related Health Problems) codes.

Most of the reviewed literature (70) used hourly or daily incremental temperature data, rather than inconsistent heat wave definitions in statistical exposure modelling. Indeed, a major criticism of many of the early studies examining gestational heat exposure was the reliance on the concept of ‘heat waves’—a term that lacks universal definitions and is subject to regional classification—both in terms of absolute temperature, and also duration [3,4]. Use of incremental temperature datapoints therefore provides ambient temperature thresholds within gestational periods, which are then used to determine fetal susceptibility, manifested by outcome variables of interest. Collection of incremental temperature datapoints also provides opportunities to study the effects of chronic heat exposure throughout gestation, reflective of climates in the global south, as well as acute heat events in both temperate and tropical/desert climates. Although more comprehensive indices of thermal discomfort exist (e.g., Universal Thermal Climate Index (UTCI; highly correlated with ambient dry temperature), Physiological Equivalent Temperature (PET), humidex), most require multiple environmental inputs and sometimes individual physiological measurements—often beyond the scope of large, retrospective population-based studies [100].

Physiological responses to ambient heat are difficult to model experimentally. Geography informs climate, with the presence of modifying elements such as elevation, topography, proximity to water, and latitudes distant from the equator associated with more temperate climates. Temperatures fluctuate diurnally, with some populations seeking relief through modern climate-control (e.g., air conditioning) or natural geographical features (e.g., subterranean, water, elevations, shade). Heat stress may be exercise—induced or passive, under low or high humidity [9,101]. In southern climates, consistently high ambient temperatures are typical, however extreme heat events occur, contributing to drought, crop failures, and loss of greenspace [102]. In more temperate regions, brief periods of days–weeks are characterized by ill-defined ‘heat waves’ [3]. Assessment of the risks of exposures to ambient temperature extremes on pregnancy are therefore challenged by concepts of acclimatation and intermittent exposures.

Heat acclimation is physiologically characterized by sudomotor responses including perspiration and increased peripheral blood flow, number and distribution of sweat glands [101]. Acute heat acclimation, as perhaps experienced during a short-term heat-wave event, or an abrupt seasonal-transition, can be achieved with brief exposure periods within days to two weeks—and is characterized by increased sweating and lowered heart rate [101]. Such acute acclimation is likely reflected in studies evaluating seasonal patterns of adverse pregnancy outcomes [103]; many studies we reviewed included the season of birth in the analysis. Although populations geographically proximal to the equator, have limited means of climate control, heat acclimation involving a reduction in sweat response, and the ability to maintain lower core and skin temperatures occurs [101]. A minority of the literature (17/84) reviewed here was set in the global south, however even with heat-acclimation, exercise, clothing or physiological states such as pregnancy can contribute to uncompensated heat stress [9,104]. Research and capacity gaps include limited epidemiological capacity and lack of maternal-birth registries, which prevent thorough investigation of gestational heat exposure and pregnancy outcomes in the global south.

### 4.1. Social Gradient of Risk

As with many environmental health risks, the social gradient of risk [105] amplifies adverse outcomes associated with gestational heat exposure in populations marginalized by poverty, racism, inequities and low socioeconomic status [7]. Socioeconomic factors contribute to ambient heat exposures through outdoor workplace settings, limited access to indoor climate control, and detrimental housing conditions in areas with elevated microenvironments due to traffic and urban density [10]. In our review, socioeconomic variables were among the demographic data reported by 45 articles, however a few articles provided comprehensive discussion about adverse pregnancy outcomes and the social gradient.

Recognizing that race is a social construct, investigation of adverse pregnancy outcomes across subpopulations can lead to identification of underlying systemic inequities and discrimination, which influence maternal comorbidities, environmental exposures and healthcare access [10,105,106]. Less than half of the reviewed studies reported race/ethnicity of pregnant people, with almost all of these studies (32/35) conducted in Australia, Europe or the United States. National records of race/ethnicity healthcare and birth registry data are not universal—such that these data may not be available for collection and analysis [106]. Race/ethnicity data was often aggregated into a priori census categories [28,73,90], emphasized Indigenous status [56,63] or used immigration status as a binary, proxy category [20,39]. In many multicultural settings, including the United States, racialized status typically reflects lower socioeconomic status [28,107]; at the intersection, this may disproportionately increase ambient gestational heat exposure. It is therefore integral to the design of gestational heat exposure studies to include race disaggregated data along with measures of socioeconomic status to best identify populations at risk of adverse pregnancy outcomes.

### 4.2. Pregnancy Outcomes

The most commonly evaluated pregnancy outcomes resulting from our review were preterm birth (44/84), birthweight (21), congenital anomalies (11) and stillbirth (10). Given the scoping review methodology, we did not evaluate the quality of each study —thus can only broadly summarize the findings. Preterm birth (<37 weeks gestation [103]), the outcome with the largest amount of literature evaluated, was generally associated with gestational heat exposure, consistent with previous meta-analyses and systematic reviews of this topic [11,12,13,108]. Several US-based studies reported greater risk of preterm birth among African American mothers [28,73,90] with several sociodemographic factors identified as determinants —such as poverty and living in mobile homes with limited climate control [90].

Biologically, the relationship between late term gestational heat exposure as a cause for preterm delivery [12] is proposed to result from maternal heat-stress/deficient thermoregulatory capacity resulting in cortisol release and subsequent oxytocin/prostaglandin-induced uterine contractions [13,28,109]. Alternatively, heat-induced modulations in maternal vascular hemodynamics may provide a parallel mechanism. Thermoregulatory studies estimate a fetal:maternal temperature difference of about 0.5 °C, owing to increased fetal metabolism [110]. When the fetus increases heat production due to metabolism and/or gestational heat exposure, thermoregulatory strategies are limited to the dissipation of heat via umbilical circulation/placenta to the maternal circulation, and towards the uterine wall [110,111]. Hyperthermia induces maternal sweating and also arginine vasopressin (AVP)-mediated renal fluid reabsorption as thermoregulatory mechanisms [112]. As AVP activates myometrium oxytocin (OTR) and vasopressin (V1aR) receptors, stimulating uterine contractions [113] this provides a plausible mechanism for heat-induced preterm labour. Fetal AVP and a related peptide, copeptin, serve as biomarkers of both acute (AVP) and cumulative (copeptin) birth stress [114,115], and may be useful as biomarkers of gestational heat stress for future studies.

In our review, birthweight was evaluated as low birthweight (<2500 g, binary variable) or reduced birthweight (continuous variable). Whereas all LBW births are either preterm or small-for-gestational age, not all preterm births are necessarily LBW [116]. Distinguishing between term-birthweight (normal weight or small-for-gestational age) and LBW due to preterm birth, requires some accuracy in the ascertainment of gestational age at birth. The literature is therefore biased towards data, which reflects standardized healthcare visits, record collection and hospital/clinic births; thereby limiting studies from low to middle-income countries where many births are unattended and may occur at home [116]. Although most studies reviewed here recognized a statistically significant negative association between gestational heat exposure and birthweight, this did not necessarily include an increase in the proportion of LBW births. Interpretation of these findings is complicated by the inclusion of both preterm and term births in statistical analyses. Exposures encompassed all three trimesters with no apparent consensus on a sensitivity period. Previous systematic reviews report a negative association of gestational heat exposure and birthweight [11], or an association with gestational cold exposure [108]. Yet another meta-analysis reported this general body of studies too heterogeneous in terms of study design and quality, and concluded that the evidence that gestational heat exposure reduces birthweight is weak [12]. Biologically, LBW can stem from a multitude of causes including maternal malnutrition, smoking, low pre-pregnancy BMI, chronic health problems, genetic or congenital anomalies and alcohol/drug use [116].

The evaluation of congenital anomalies as an outcome of ambient gestational heat exposure is complicated by the heterogeneity of this category of adverse pregnancy outcomes. Consistent with fetal development, the included studies (11) primarily evaluated gestational heat exposures during the first trimester—capturing primary morphogenesis, and early organogenesis [117]. To ensure accurate congenital anomaly case numbers, data should include term and preterm births, stillbirths and elective terminations, biasing data collection to hospital–clinical records [118]. Further, cases with genetic and chromosomal anomalies should be excluded in studies designed to evaluate purely environmental etiologies [119], however, such study designs will be limited to healthcare settings with advanced technologies in prenatal imaging, genetic testing, and fetal/pediatric pathology. Congenital heart disease represented the most common category of congenital anomalies reviewed here, however in many studies this was a heterogeneous grouping of septal defects, Tetralogy of Fallot and conditions related to vascular transposition. Activation of two heat-sensitive ion channels (TRPV1 and TRPV4) in chick cardiac neural crest cells—one of four cardiac progenitor cell types—produce hyperthermic-cardiac defects [120], and provide a possible mechanism for maternal hyperthermia caused by fever or ambient heat exposure [119]. Although studies included in our review described potential associations of gestational heat exposure and non-cardiac congenital anomalies (e.g., neural tube defects, orofacial clefts, hypospadias), future systematic evaluation should limit outcome variables to specific congenital defects, perhaps prioritizing investigations of cardiac anomalies due to the preponderance of studies available.

The final category of pregnancy outcomes—stillbirth—was consistently defined as fetal loss over 20 weeks gestation [121]; once again, term and preterm exposures were often analyzed together. Generally, our ten reviewed studies reported positive associations between stillbirth and gestational heat exposure during any part of pregnancy. Although this is generally consistent with previous reviews [11,12], the paucity of studies examining stillbirth as an outcome requires caution in the interpretation of these findings. Multifactorial events may culminate in stillbirth including combinations of genetic/chromosomal abnormalities, congenital anomalies, placenta pathologies, infections and umbilical cord complications [121]. The “triple risk model” [122] builds on a multifactorial etiology of adverse outcomes, wherein the fetus is made vulnerable by an underlying genetic/chromosomal or congenital anomaly, and at a sensitive window of development an exogenous stressor such as maternal ambient heat exposure is presented [121]. Identification of gestational heat exposure as a cause of stillbirth will require highly integrated analyses of maternal and fetal stressors, exposure windows and possibly, multiple adverse outcomes.

### 4.3. Limitations

Inherent to the design of scoping reviews, we did not assess the methodological quality of the included papers, and so we discuss only general, albeit, limited findings regarding gestational heat exposure and adverse pregnancy outcomes. Although we have critiqued the body of literature as deficient in studies set in the global south, this may be due to our restriction to those studies published in English. We also acknowledge that attempts to present study characteristics such as design, setting, statistical analysis and gestational exposure may have obscured outliers from our categorical approach.

## 5. Conclusions

The frequency and severity of extreme heat events due to climate change will continue to impact the global health of pregnant people. This scoping review complements existing systematic reviews/meta-analyses, which evaluate gestational heat exposure and adverse pregnancy outcomes. Despite heterogeneity in study design and sample size, temperature measurements and adverse pregnancy outcome case definitions in this literature are rigorous, enabling future comparative studies. Gaps in this field include a paucity of studies from the global south, and more comprehensive investigation of individual adverse pregnancy outcomes beyond preterm birth. Similarly, the impacts of systemic racism and poverty on gestational heat exposure due to housing, neighborhood and workplace disparities were often not meaningfully integrated with study conclusions, despite a collection of socioeconomic indicators. Finally, although these epidemiological studies associate gestational heat exposure with adverse pregnancy outcomes, a greater understanding of the biological mechanisms underlying these relationships is needed.

## Figures and Tables

**Table 1:** Study characteristics *n* = 84.

	Count	%
Year of Publication		
2010–2015	20	24
2016–2020	64	76
Study Setting ^1^		
Australia	6	7
Eastern Europe	1	1
Northern Europe	2	2
South America	2	2
Southern Europe	10	12
Western Asia	5	6
Western Europe	5	6
East Asia	16	19
Northern America	34	40
West Africa	2	2
Start Dates of Data Collection/Inclusion ^2^		
1931–1949	1	1
1950–1974	3	4
1975–1999	35	42
2000–2020	46	55
Duration of Data Collection/Inclusion ^3^		
less than 5 y	22	26
5–9 y	28	33
10–14 y	18	21
15–19 y	4	5
20–24 y	6	7
25+ y	7	8

^1^ Kwag et al. [55] was reviewed as an Epub preprint (17 December 2020), subsequently published in 2021. ^2^ Davenport et al., 2020 comprised of multiple African Demographic and Health Survey (DHS) from 15 African countries wherein each DHS cycle was characterized by different survey dates (start, duration) [40]. Jensen and Sørensen, 2013 comprised of global birthweight/climate comparisons from over 60 countries [50]—not included in the table. ^3^ Schifano et al., 2016 (Rome, Barcelona [72]) and Wolf and Anderson, 2012 (Brandenburg, Saxony [92]) each consist of two data collection regions each with a different ^4^ duration.

**Table 2:** Summary of study methodologies *n* = 83.

	Count	%
Study Design		
Case-Control	7	8
Case-Crossover	11	13
Case-Series	2	2
Cohort	31	37
Cross-sectional	3	4
Population Registry	16	19
Time-series, Registry	13	15
Not Stated	1	1
Statistical Analysis ^1^		
Case-Crossover Analysis	6	7
Fixed Effect-Poisson Distribution	16	19
General Linear Model	9	11
Pearson Correlation	2	2
Regression Models	34	40
Survival Analysis-Time	30	36
Temperature Measurements		
Hourly ^2^	7	8
Daily	63	75
Monthly	5	6
Seasonal	1	1
Climate ^3^	2	2
Heat wave	6	7
Exposure Period		
1st Trimester	12	14
2nd + 3rd Trimester ^4^	15	18
3rd Trimester	8	10
All/Any Part of Pregnancy ^5^	35	42
Delivery Date	13	15

^1^ Several studies used multiple statistical approaches, such that the total exceeds 84 included papers. ^2^ Agay-Shay et al., 2013, provided temperature data in 0.5 h increments [15]. ^3^ Temperature was classified using climatic patterns for evaluation of US counties by Carmichael et al., 2014 [33], and 60 countries by Jensen and Sørensen, 2013 [50]. ^4^ Multiple studies included temperature exposures up to month prior to delivery in analysis; included in 2nd/3rd trimester. ^5^ Molina et al., 2017 used monthly exposures throughout pregnancy in analysis [65], Li et al., 2018, Martens et al., 2019 used weekly exposures in analysis [56,57,62]; and Ha et al., 2017 included multiple exposure periods from 1st/2nd trimesters in analysis [44]—included in any part of pregnancy.

**Table 3:** Preterm Birth Study Characteristics *n* = 44.

	Count	%
Study Design		
Case-Crossover	5	11
Case-Series	1	2
Cohort	17	39
Population Registry	10	23
Time-Series, Registry	11	25
Setting		
Australia	5	11
Eastern Asia	12	27
Eastern Europe	1	2
Northern America	14	32
Northern Europe	1	2
Southern Asia	1	2
Southern Europe	7	16
Western Asia	1	2
Western Europe	2	5
Exposure Period		
Conception	1	2
2nd + 3rd Trimester ^1^	11	25
3rd Trimester	7	16
All/Any Part Of Pregnancy ^2^	13	30
Delivery Date	12	27

See reference list for 44 studies included in analysis [17,19,20,24,25,28,31,33,35,37,42,43,44,45,48,52,54,55,57,58,63,64,66,67,71,72,73,76,79,80,81,83,84,85,86,87,88,90,91,92,93,96,97,98]. ^1^ Several studies included temperature exposures up to month prior to delivery in analysis; included in 2nd/3rd trimester. ^2^ Ha et al., 2017, included multiple exposure periods from 1st/2nd trimesters in analysis [44]—included in any part of pregnancy.

**Table 4:** Birthweight Study Characteristics *n* = 21.

	Count	%
Study Design		
Cohort	10	48
Cross-sectional	2	10
Population Registry	4	19
Time-series, Registry	4	19
Not stated	1	5
Setting		
Africa	1	5
Australia	1	5
Eastern Asia	4	19
Global	1	5
Northern Europe	1	5
Southern America	4	19
United States	4	19
Western Asia	1	5
Western Europe	4	19
Exposure Period		
All/Any Part of Pregnancy ^1^	17	86
Delivery Date	3	14

See reference list for 21 included studies [16,32,34,39,40,41,47,49,50,54,56,59,61,65,66,68,69,76,91,92,94]. ^1^ Molina et al., 2017, used monthly exposures throughout pregnancy in analysis [65], Li et al., 2018, used weekly exposures in analysis [56]—included in any part of pregnancy.

**Table 5:** Congenital Anomaly Study Characteristics *n* = 11.

	Count	%
Study Design		
Case-Control	7	64
Case-Crossover	1	9
Cohort	3	27
Setting		
Canada	2	18
Israel	1	9
Turkey	1	9
United States	7	64
Exposure Period		
1st Trimester	10	91
All/Any Part of Pregnancy	1	9
Type of Congenital Anomaly ^1^		
Congenital Heart Defect	5	45
Hypospadias	1	9
Neural Tube Defect	2	18
Orofacial Clefts	1	9
Various	2	18

^1^ Most studies were designed to evaluate specific congenital anomalies (congenital heart defects [15,22,60,78,95], neural tube defects [23,74], hypospadias [53], orofacial clefts [75]), with two studies which assessed multiple anomalies (“various”) [29,82].

**Table 6:** Stillbirth Study Characteristics *n* = 10.

	Count	%
Study Design		
Case-Crossover	4	40
Cohort	2	20
Cross-Sectional	2	20
Population Registry	2	20
Setting		
Australia	3	30
Canada	1	10
Sub-Saharan Africa	2	20
Taiwan	1	10
United States	3	30
Exposure Period		
1st Trimester	1	10
2nd/3rd Trimester	2	20
3rd Trimester	1	10
Delivery Date	1	10
All/Any Part of Pregnancy ^1^	5	50

See reference list for included studies [18,21,30,40,51,57,70,79,87,91]. ^1^ Li et al., 2018, used weekly exposures in analysis [57]—included in any part of pregnancy.

## Data Availability

Studies which comprised this scoping review are listed as references #15-98.
